# Detection of Ricin Contamination in Ground Beef by Electrochemiluminescence Immunosorbent Assay

**DOI:** 10.3390/toxins3040398

**Published:** 2011-04-04

**Authors:** David L. Brandon

**Affiliations:** Foodborne Contaminants Research Unit, Western Regional Research Center, USDA Agricultural Research Service, 800 Buchanan Street, Albany, CA 94710, USA; Email: david.brandon@ars.usda.gov; Tel.: +1-510-559-5783; Fax: +1-510-559-5880

**Keywords:** ricin, *Ricinus communis* agglutinin, castor, monoclonal antibody, biothreat, electrochemiluminescence

## Abstract

Ricin is a highly toxic protein present in the seeds of *Ricinus communis* (castor), grown principally as a source of high quality industrial lubricant and as an ornamental. Because ricin has been used for intentional poisoning in the past and could be used to contaminate food, there is a need for analytical methodology to detect ricin in food matrices. A monoclonal antibody-based method was developed for detecting and quantifying ricin in ground beef, a complex, fatty matrix. The limit of detection was 0.5 ng/g for the electrochemiluminescence (ECL) method and 1.5 ng/g for enzyme-linked immunosorbent assay (ELISA). The detection of nanogram per gram quantities of ricin spiked into retail samples of ground beef provides approximately 10,000-fold greater sensitivity than required to detect a toxic dose of ricin (>1 mg) in a 100 g sample.

## 1. Introduction

The detection of naturally occurring toxins and the validation of test methods in food matrices are needed to protect consumers from both adventitious and intentional adulteration of foods. Ricin is a highly toxic protein found in the seeds (beans) of the castor plant, *Ricinus communis*, and consists of two chains of about 32 kDa, joined by a single disulfide bond (see reviews [1,2]). Although ricin would not be expected to contaminate foodstuffs naturally, there has been concern over the potential contamination of the food supply with ricin as an act of bioterrorism [3]. Indeed ricin has been used maliciously in the past and has been found at a number of locations as a result of apparent criminal activity, e.g., [4]. For these reasons, it is important to have sensitive methods for detecting ricin and marker compounds associated with crude ricin preparations. 

Although animal models provide irrefutable means for quantifying toxins encountered enterically [5], especially crude toxins in complex food matrices, immunochemical tests for structural determinants and mechanism-based assays for activity can also provide essential analytical data for food safety assurance, e.g., [6,7]. A number of in vitro assays have been developed for ricin, including immunoassays [8,9,10,11], activity assays [6,12], immunochromatographic devices [13], and other array and sensor technologies [14,15,16]. In addition, assays that measure compounds found in crude preparations of the toxin, such as castor DNA [17,18] and the alkaloid ricinine [19], offer additional means of detection and forensic attribution. Immunochemical technology has also been combined with the prodigious amplification potential of PCR to develop an exquisitely sensitive immuno-PCR assay for ricin [7]. 

Electrochemiluminescence (ECL) detection is a promising technology that exploits multiple excitation cycles to amplify the luminescent signal and improve sensitivity. The mechanism of excitation and the relatively long emission wavelength (620 nm) potentially provide resistance to matrix effects. An ECL microplate method for quantifying ricin B chain was described by Guglielmo-Viret and Thullier [20]. Garber and O’Brien [21] have also described ECL immunosorbent methodology for detecting the ricin molecule in a variety of beverages, using monoclonal and polyclonal antibodies. Cho *et al.* [22] used ECL technology in a plate format for an activity assay of ricin in a variety of liquid food matrices. Nevertheless, solid, fatty matrices such as ground beef remain challenging. In addition, the “hook effect” in the dose-response curve makes some samples more difficult to analyze, requiring multiple dilutions for quantification [21,23].

In this study, electrochemiluminescence was evaluated as a detection method for ricin in ground beef, in comparison with enzyme-linked immunosorbent assay (ELISA). Multi-well ECL plates were used, coated by adsorption with a single ligand (a mouse monoclonal antibody), analogous to standard 96-well ELISA plates, and the two assay formats were compared. 

## 2. Materials and Methods

### 2.1. Homogenizer

Model GLH-01 homogenizer with a 10 mm × 115 mm, saw tooth generator probe (Omni International, Kennesaw, GA, USA) was generally used at ca. 20,000 rpm (#8 setting of Omni GLH external Speed Control SC115). Initial studies utilized the lighter duty Omni model TH-01 homogenizer, but this model proved unable to maintain speed with some samples.

### 2.2. Samples

Ground beef marked “90% lean” was purchased at a local supermarket and used within 24 h of purchase. Samples were kept on ice during all procedures prior to application of sample to assay wells. Four-gram samples were weighed into 50 mL polypropylene conical centrifuge tubes and spiked with a small volume (generally 8 µL) of ricin solution. Each sample was thoroughly mixed using a plastic spatula, and then 8 mL of extraction buffer were added (phosphate-buffered saline [PBS]-100 mM galactose). Samples were homogenized for 30 s at 20,000 rpm, and pieces of beef were then dislodged from the homogenizer probe and returned to the homogenate using a spatula. The sample was homogenized for an additional 30 s at the same speed. Between samples, the probe was cleaned by two washes with water at 30,000 rpm. Deionized water was used for all washes and buffers in this study.

### 2.3. Toxins

Ricin and RCA-1 were obtained from Vector Laboratories (Burlingame, CA, USA). For preparation of crude ricin (CR), castor seeds were weighed and ground thoroughly in mortar and pestle, in PBS (10 mL/g). The homogenate was centrifuged at 3000 g for 5 min in a fixed angle rotor, and the aqueous supernatant, below the oil layer, was collected. This procedure was repeated 3 additional times. The protein content was determined by assay with bicinchoninic acid [24], and ricin and RCA-1 were estimated as 2.4 mg/mL and 3.0 mg/mL, respectively, by ELISA [11]. The extract was diluted to 1 mg/mL ricin and used to spike ground beef samples.

### 2.4. Assay Plates

Colorimetric ELISAs were performed on Immulon^®^ 4HBX plates (Dynex, Chantilly, VA, USA), coated as described previously [11]. Briefly, wells were coated with proteins at 5 μg/mL in PBS, excess “sticky” sites were blocked with 10 mg/mL BSA in PBS-0.05% Tween^®^-20 (BPT). Coated, rinsed plates were treated with 2% sucrose, then dried at 37 °C and stored desiccated at 4 °C for up to 6 months. For ECL assays, 96-well standard, uncoated plates were obtained from Meso Scale Discovery ([MSD], Gaithersburg, MD, USA; Cat. No. L15XA-3). Details for antibody coating are given below. 

### 2.5. Antibodies and Conjugated Antibodies

Monoclonal antibodies were prepared using isolated ricin A and B chains as immunogens in BALB/c mice. MAbs were purified, characterized, and biotinylated, as described previously [11]. Tris(2,2’-bipyridyl)ruthenium(II) (Ru[bpy]_3_)-conjugates of antibodies were prepared using the *N*-hydroxysuccinimide ester (MSD, Cat. No. R91AN-1) and spin columns for buffer exchange and conjugate purification, per manufacturer’s protocol (Sulfo-Tag^®^ labeling kit, MSD, Cat. No. R91CN-1). MAbs are designated by the corresponding hybridoma clone numbers, prefixed by designation of the conjugate, for example b-1795 for biotinylated mAb (b-mAb) 1795 and Ru-1443 for Ru(bpy)_3_-conjugated mAb 1443.

### 2.6. ELISA Conditions

Assay wells contained 100 μL of standards, controls, or samples, using BPT containing 100 mM galactose as diluent. Samples were generally assayed neat or as dilutions of 1:2, 1:5, or 1:10 by addition 100, 50, 20 or 10 μL of sample to assay wells containing 0, 50, 80, or 90 μL, respectively, of BPT-galactose. After samples and standards were applied, plates were sealed and incubated 1 h, with shaking. Wells were emptied and rinsed by manual pipetting of wastes into 0.5% sodium hypochlorite solution for inactivation of toxin, and wells were rinsed an addditional 4 times with water. Biotinylated detection antibody was then added (100 μL at 100 ng/mL in BPT containing 100 mM galactose, to minimize nonspecific binding and binding to agglutinins via their carbohydrate-binding sites). After incubation with shaking for 1 h, wells were washed 4 times with water. Horseradish peroxidase (HRP)-conjugated streptavidin (Invitrogen, South San Francisco, CA, USA) was applied (1:5000, 100 μL/well) and incubated 30 min, with shaking. Following water washes, the assay was developed by adding tetramethylbenzidine substrate solution (TMB, K-Blue, Neogen, Lexington, KY, USA), 100 μL/well. The reaction was stopped after 30 min by the addition of 100 µL/well 0.3 N HCl. Absorbance was read at 450 nm, with subtraction of the absorbance at 650 nm, using Model M2 plate reader using SoftMax^®^ Pro 5.3 software (Molecular Devices, Sunnyvale, CA, USA). 

### 2.7. ECL Assay Conditions

In these assays, all steps were conducted with a total volume of 30 μL, except for application of biotinylated mAbs which employed 50 μL. Dilutions were prepared as for the ELISA described above, with scaling for volume. ECL wells were washed 4 times between steps with PBS-0.05% Tween-20 (PBST). Incubations were 60 min for application of samples and standards, 30 min for other steps. 

### 2.8. ECL Assay Using mAb-Coated Assay Plates

In sandwich assays employing biotinylated detection antibodies, the secondary reagent was streptavidin conjugated with Ru(II) tris-bipyridine 4-methylsulfonate (MSD Sulfo-Tag—Streptavidin, Cat. No. R32AD-5), 30 µL/well at 0.5 µg/mL diluted in BPT-100 mM galactose. For assays utilizing direct detection of analyte, Ru(bpy)_3_-conjugated mAbs were used (50 μL/well, 0.2–1 μg/mL). Plates were again washed 4 times, as above, tapped to empty, then 150 µL of tripropylamine solution (MSD Read Buffer with Surfactant, Cat. No. R92TC-2, diluted 1:4 with water) were added. After dispersing any bubbles that formed during pipetting, plates were read immediately on a 2400 Sector 2400 Imager, with Discovery Workbench v3.0 software (MSD). 

### 2.9. Data Analysis

Data are expressed as mean ± standard deviation (sd, *n *= 3), unless otherwise indicated. Analyses of ground beef were done at least 5 times, with results shown from one typical experiment. Limit of detection (LOD) was computed as the analyte concentration at which the lower one-sided 95% confidence interval (CI) equaled the blank + 3 sd. Confidence and prediction intervals were computed using SlideWrite^®^ v6 (Advanced Graphics Software, Carlsbad, CA, USA). Assay curves were fitted to a 4-parameter logistic model using either SlideWrite or SoftMax Pro. 

## 3. Results

### 3.1. Capture and Detection Antibody Concentrations

**Figure 1:**
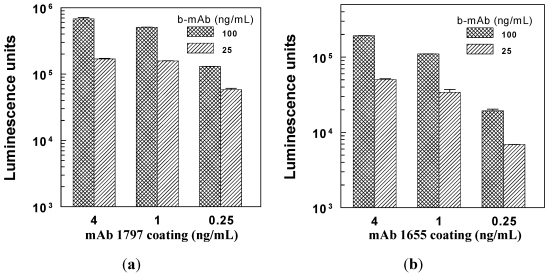
Capture and detection antibody conditions on standard ECL plates. (**a**) Detection mAb 1443, capture mAb 1797. Average backgrounds were 303 and 333 for detection with 25 and 100 ng/mL biotinylated mAb, respectively; (**b**) Detection mAb 2147, capture mAb 1655. Average backgrounds were 410 and 335 for detection with 25 and 100 ng/mL biotinylated mAb, respectively.

**Figure 2:**
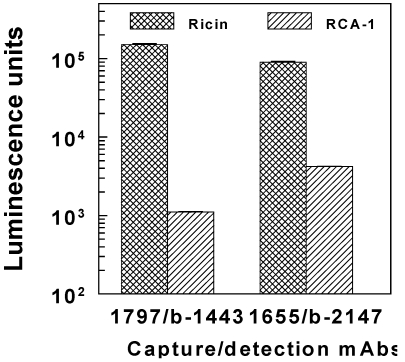
Discrimination between ricin and RCA-1 by two sandwich pairs in standard ECL assay, with secondary detection by Ru(bpy)_3_-streptavidin. Luminescence readings are shown for 100 ng/mL of agglutinin, with blanks subtracted (mean ± sd).

For directly coated ECL plates, a variety of conditions were tested. Figure 1 shows results obtained using 2 different antibody pairs. As expected, signal increased at higher coating concentrations and higher b-mAb concentrations. Luminescence was 300–400% greater with mAb coated at 1 μg/mL instead of 0.25 μg/mL, but increased less than 100% when the plate coating concentration was increased to 4 μg/mL. The use of 100 μg/mL b-mAb increased the ECL signal by about 200% compared to the results obtained at 25 μg/mL. Standard conditions selected for subsequent studies were 2 µg/mL for coating the capture mAbs and 100 ng/mL for biotinylated detection mAbs. The ability of these antibody pairs to discriminate between ricin and RCA-1 is illustrated in Figure 2. The 1797/b-1443 pair afforded 2 orders of magnitude selectivity in the detection of ricin over RCA-1 at the highest level tested (100 ng/mL), and greater selectivity was shown at lower concentrations of analytes. 

**Figure 3:**
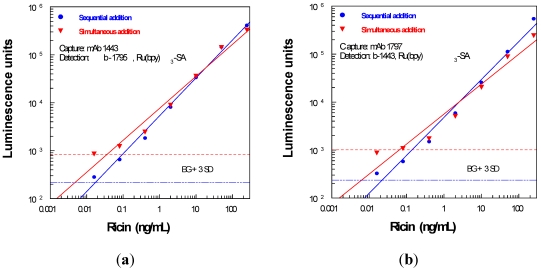
Two modes of indirect detection of biotinylated detection mAbs. (**a**) Assay with capture mAb 1443; (**b**) Assay with capture mAb 1797. In each case, the background (BG) + 3 sd is indicated by the broken line, with the upper (dashed) line corresponding to the simultaneous addition of detection reagents, b-mAb + Ru(bpy)_3_-streptavidin.

To shorten assay time, it could be advantageous to add b-mAb and Ru(bpy)_3_-streptavidin simultaneously, rather than performing separate incubation and wash steps for the two reagents. Figure 3 illustrates the results of comparing procedures. Simultaneous reagent addition raised the background ECL approximately 5-fold, apparently due to nonspecific binding of the b-mAb-streptavidin complex to the capture mAb layer. Although the higher background only influenced the ECL response significantly below 1 ng/mL ricin, all further assays employed sequential addition. 

### 3.2. Directly Labeled mAbs for Detection

**Figure 4:**
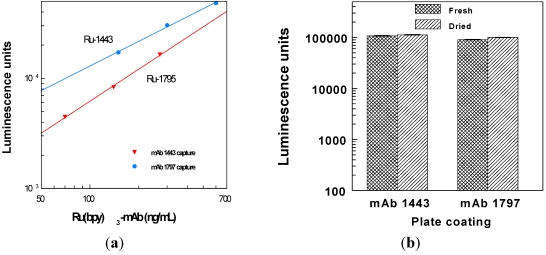
(**a**) Titration of Ru-1795 and Ru-1443 on standard ECL-coated plates; (**b**) Assays with b-mAb/Ru(bpy)_3_-streptavidin were conducted on freshly coated plates, as well as on plate coatings stabilized with sucrose after blocking, dried at 37 °C (90 min), and stored desiccated overnight.

An alternative assay format used directly labeled Ru(bpy)_3_-mAbs, instead of indirect detection of biotinylated mAbs using streptavidin conjugate. The titration of two labeled mAbs is illustrated in Figure 4(a). The useful assay range (>10^4^ luminescence units) corresponded to labeled mAbs at approximately 200 ng/mL. Another assay parameter that was investigated was the performance of fresh *versus* dried assay plates. As shown in Figure 4(b), assays conducted on plates freshly coated with mAb were indistinguishable from those conducted on dried mAb-coated plates. Because the two coating protocols produced similar results, dried plates were used in subsequent experiments. This facilitated work flow and enabled preparation of batches of coated plates, for most reproducibility.

### 3.3. Recovery of Ricin from Ground Beef

Ground beef was spiked with pure or crude ricin and homogenized in PBS-galactose (2 mL/g). The slurry was diluted for application to mAb-coated assay wells. 

### 3.4. Purified Ricin

**Figure 5:**
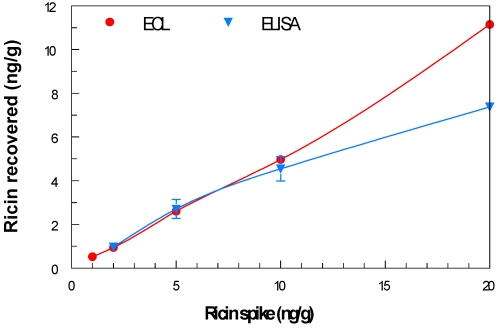
Analysis of ground beef spiked with pure ricin from 1–20 ng/g by ECL and ELISA. Side-by-side assays used mAb 1443 coated by adsorption on standard assay plates and detected using b-1795 and conjugated streptavidin.

**Figure 6:**
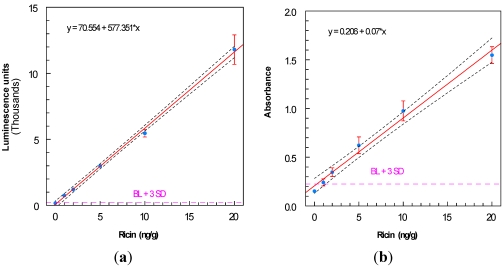
LOD determination for (**a**) ECL analysis (0.5 ng/g), and (**b**) ELISA (1.5 ng/g), both using mAb 1443 for capture, b-1795 + conjugated streptavidin for detection. In each graph, the lower 95% one-sided confidence interval is indicated by the dotted line below the fitted standard curve, for which the linear equation is shown. Blanks were 165 ± 9.8 luminescence units for ECL and 0.152 ± 0.024 absorbance units for ELISA. The dashed lines indicate the blank + 3 sd.

Figure 5 illustrates the recovery of pure ricin from ground beef, determined by ELISA as well as ECL analysis. Recoveries varied from 30 to 60% at different spike levels, but were more consistent and generally higher by ECL analysis. The 1 ng/g spike could not be determined quantitatively by ELISA, but was readily determined by ECL. The limit of detection was also computed for each assay (Figure 6): 0.5 ng/g for ECL and 1.5 ng/g for ELISA.

### 3.5. Crude Ricin

**Figure 7:**
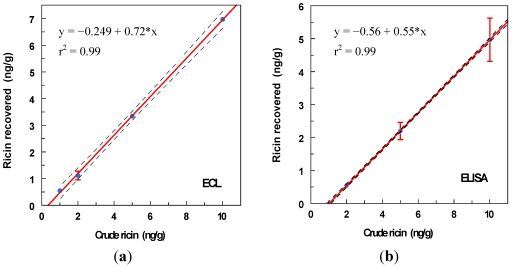
Ground beef spiked with crude ricin prepared from castor seeds was analyzed by (**a**) ECL assay and (**b**) ELISA. The 95% confidence interval on the linear regression is shown.

The analysis of ground beef spiked with crude ricin is illustrated in Figure 7. 

## 4. Discussion

Parallel assays in ECL and ELISA formats were conducted in standard mAb-coated 96-well ELISA plates, using biotinylated mAb and conjugated streptavidin for detection. The typical 96-well format is familiar to users of ELISA technology. Both ELISA and ECL can be used for detection with adequate sensitivity in the nanogram per gram range. Spiking of ground beef simulated conditions that might be expected in an intentional contamination incident, and employed a small volume of soluble purified or crude ricin (e.g., castor bean aqueous extract). Detection is possible orders of magnitude below toxic levels. Although acute toxicity could be caused by 1 mg of ricin, the estimated lethal dose by ingestion is substantially higher [25,26]. The described assays could detect 100 ng evenly dispersed in a typical raw hamburger patty. Furthermore, ricin, although relatively thermostable, is substantially inactivated by conditions used to cook ground beef safely [6].

In highly multiplexed versions of commercialized ECL technology, 96-well plates can have up to 25 spots—patterned arrays of immobilized ligand—permitting thousands of assays in a single plate. Compared to ELISA technology, ECL instrumentation and reagents are relatively expensive, with disposable costs about 10-fold higher. However, highly multiplexed formats, although not used in this study, would greatly reduce the cost per assay. Coupling of the Ru(bpy)_3_ *N*-hydroxysuccinimide ester to antibody is performed as readily and stably as with more familiar biotin esters, offering resistance to conditions that could interfere with or inactivate enzymes.

ELISA offers the ease of a more familiar assay system, with relatively inexpensive materials. Even without instrumental reading, qualitative results can be evaluated visually, though with reduced sensitivity (ca. 10 ng/g). In contrast, ECL offers a wider dynamic range of quantitative determination, with lower coefficients of variation (4% within assay *versus* 12% for ELISA), and less interference from complex, fatty food matrices such as ground beef. Both assays produced relatively low blanks with unspiked ground beef samples (shown, for example, in Figure 7), but the signal for 10 ng/mL was 30-fold higher than the blank for ECL, compared to 6-fold for ELISA. One hundred mM galactose was routinely included in assay buffers to minimize interaction of the castor agglutinins with the carbohydrate of antibodies. Other approaches, such as the use of antibody fragments [27] or single-domain antibodies [28] lacking the carbohydrate-rich Fc region, are alternatives that could prove useful in lowering assay blanks or matrix effects for some food analytes.
